# Ukrainian refugees in Germany – what are the consequences for mental health care services in Germany?

**DOI:** 10.1192/j.eurpsy.2023.112

**Published:** 2023-07-19

**Authors:** I. T. Graef-Calliess

**Affiliations:** Hannover Region Clinics, Hannover, Germany

## Abstract

**Abstract:**

Since about a year there has been an influx of refugees from Ukraine due to the current war situation.

Data on the mental health condition of Ukrainian refugees will be reviewed and compared to the mental health conditions to refugees from other parts of the world in Germany. Moreover, the psychosocial and socioeconomic situation of refugees from Ukraine will be reflected on the background of the situation of refugees from other countries.

Conclusions from the recent experiences for the mental health care system will be drawn and discussed with the aufience in a European perspective. Special highlight will be given to pilot best practice modells for mental health care of Ukrainian refugees within the German mental health care system.

**Disclosure of Interest:**

None Declared

